# Development of Improved HDAC6 Inhibitors as Pharmacological Therapy for Axonal Charcot–Marie–Tooth Disease

**DOI:** 10.1007/s13311-016-0501-z

**Published:** 2016-12-12

**Authors:** Veronick Benoy, Pieter Vanden Berghe, Matthew Jarpe, Philip Van Damme, Wim Robberecht, Ludo Van Den Bosch

**Affiliations:** 1 KU Leuven, Department of Neurosciences, Experimental Neurology and Leuven Research Institute for Neuroscience and Disease (LIND), University of Leuven, B-3000 Leuven, Belgium; 2 VIB, Center for Brain and Disease Research, Laboratory of Neurobiology, B-3000 Leuven, Belgium; 3 Translational Research Center for Gastrointestinal Disorders (TARGID), University of Leuven, B-3000 Leuven, Belgium; 4 Acetylon Pharmaceuticals Inc., Boston, MA USA; 5 Department of Neurology, University Hospitals Leuven, B-3000 Leuven, Belgium

**Keywords:** Charcot–Marie–Tooth disease, HSPB1, Histone deacetylase 6, Mitochondria, Acetylated α-tubulin, Compound screening

## Abstract

Charcot–Marie–Tooth disease (CMT) is the most common inherited peripheral neuropathy, with an estimated prevalence of 1 in 2500. The degeneration of motor and sensory nerve axons leads to motor and sensory symptoms that progress over time and have an important impact on the daily life of these patients. Currently, there is no curative treatment available. Recently, we identified histone deacetylase 6 (HDAC6), which deacetylates α-tubulin, as a potential therapeutic target in axonal CMT (CMT2). Pharmacological inhibition of the deacetylating function of HDAC6 reversed the motor and sensory deficits in a mouse model for mutant “small heat shock protein B1” (HSPB1)-induced CMT2 at the behavioral and electrophysiological level. In order to translate this potential therapeutic strategy into a clinical application, small drug-like molecules that are potent and selective HDAC6 inhibitors are essential. To screen for these, we developed a method that consisted of 3 distinct phases and that was based on the pathological findings in the mutant HSPB1-induced CMT2 mouse model. Three different inhibitors (ACY-738, ACY-775, and ACY-1215) were tested and demonstrated to be both potent and selective HDAC6 inhibitors. Moreover, these inhibitors increased the innervation of the neuromuscular junctions in the gastrocnemius muscle and improved the motor and sensory nerve conduction, confirming that HDAC6 inhibition is a potential therapeutic strategy in CMT2. Furthermore, ACY-1215 is an interesting lead molecule as it is currently tested in clinical trials for cancer. Taken together, these results may speed up the translation of pharmacological inhibition of HDAC6 into a therapy against CMT2.

**Electronic supplementary material:**

The online version of this article (doi:10.1007/s13311-016-0501-z) contains supplementary material, which is available to authorized users.

## Introduction

Post-translational modifications are important regulators of protein function. Histone acetyltransferases add acetyl groups to the lysine residues of histones, thereby altering the structure of chromatin and changing gene expression, and DNA replication and repair [1]. Removal of the ε-N-acetylated lysine residue of histones is catalyzed by the histone deacetylases (HDACs). The latter family comprises 11 enzymes (HDACs 1–11), based on the homology with the yeast HDACs, wherein 4 main classes of HDACs can be distinguished: class I (HDACs 1, 2, 3, and 8), class IIa (HDACs 4, 5, 7, and 9), class IIb (HDACs 6 and 10), and class IV (HDAC11). Apart from the zinc-dependent HDACs, the nicotinamide dehydrogenase-dependent enzymes are grouped as class III HDACs and comprise the sirtuins (1–7) [2]. Although histones are the most studied substrates of HDACs, there are also a wide variety of nonhistone substrates, including transcription factors, cytoskeletal proteins, metabolic enzymes, and chaperones [3]. Therefore, HDACs are often referred to as lysine acetylases to better indicate their substrate specificity [4].

HDAC6 functions as the major deacetylating enzyme of α-tubulin [5], and is the only member of the HDAC family to contain a C-terminal zinc-finger ubiquitin-binding domain together with 2 catalytic domains at the N-terminus. Acetylation of α-tubulin is important for the binding of molecular motor proteins (kinesin family and dynein) to the microtubules [6, 7]. Hence, decreased acetylation is suggested to influence axonal transport dynamics. Disturbances in axonal transport are often associated with peripheral neuropathies [8]. Moreover, HDAC6 and alterations in α-tubulin acetylation have been associated with several human disorders such as Alzheimer’s disease, Parkinson’s disease, Huntington’s disease, amyotrophic lateral sclerosis, and Charcot–Marie–Tooth disease (CMT) [9–13].

CMT is the most common inherited peripheral neuropathy, characterized by the progressive length-dependent degeneration of motor and sensory nerve axons. Currently, there is no therapy available for these patients [14]. In total, mutations in > 50 genes have been associated with either the demyelinating form (CMT1) or the axonal form (CMT2) of CMT [15]. Mutations in the small heat shock protein B1 (HSPB1) gene constitute one of these underlying genetic causes, leading to CMT2F in patients [16, 17]. Previously, we developed and characterized a mouse model for mutant HSPB1-induced CMT which overexpresses either wild-type or mutant human cDNA of HSPB1 in neurons [13]. Typically, these mice develop motor problems and sensory deficits from the age of 6 months on that are reminiscent to CMT2F in human patients. These defects in motor and sensory function are due to the progressive degeneration of motor and sensory nerve axons, demonstrated by reduced compound muscle action potentials (CMAPs) and sensory nerve action potentials (SNAPs). At the pathologic level, we found decreased levels of acetylated α-tubulin in sciatic nerve [13]. In addition, the axonal transport of mitochondria was disturbed in cultured dorsal root ganglion (DRG) neurons from symptomatic mutant HSPB1 mice. Interestingly, pharmacologic inhibition of the deacetylating function of HDAC6 not only normalized the defects seen in cultured DRG neurons, but also reversed the motor and sensory abnormalities and the electrophysiological defects observed *in vivo* [13].

HDAC6 is an attractive target in the development of a pharmacological therapy as mice with genetic deletion of HDAC6 are viable and have a normal life span. Moreover, research focusing on the development of several small-molecule inhibitors with improved pharmacokinetic properties (e.g., stability and blood–brain barrier permeability) is expanding [18–21]. Already a plethora of HDAC antagonists exist, all of which have made their way to the market but lack isoform selectivity. Hydroxamic acids like suberoylanilide hydroxamic acid (SAHA, also known as varinostat), a Food and Drug Administration-approved drug, and trichostatin A (TSA) are pan-HDAC inhibitors with affinity for both class I and II HDACs [22]. The first HDAC6-specific inhibitor, tubacin, was discovered in 2003 through a multidimensional, cell-based screening of a deacetylase-biased 1,3-dioxane library [23]. However, useful as a research tool, tubacin was not favorable as a drug-like compound because of its high lipophilicity, fast metabolization *in vivo*, and tedious synthesis. In pursuit of high-selective and drug-like inhibitors of HDAC6, tubastatin A, a hydroxamic acid, was developed based on structural homology modeling showing inhibitory capacity both *in vitro* and *in vivo* [24]. Continuing from this mile stone in the development of potent and selective inhibitors of the deacetylating function of HDAC6, we set up a screen to identify new small molecules with improved pharmacological properties in the exploration of a potential pharmacological therapy for CMT.

The goal of this study was to identify potent and selective inhibitors of the deacetylating function of HDAC6 as a potential pharmacological therapy for CMT. Based on the pathological and behavioral deficits found in the mutant HSPB1^S135F^ CMT2 mouse model, a study was conducted which consisted of 3 distinct phases in which the candidate HDAC6 inhibitors were tested for their potency and selectivity to inhibit HDAC6. In a first step, the effect on the acetylation of α-tubulin and histones was assessed in a neuronal cell line. Second, we evaluated these inhibitors for their efficiency to restore the mitochondrial axonal transport defects in cultured DRG neurons from symptomatic mutant HSPB1 mice. Finally, these HDAC6 inhibitors were tested in the mutant HSPB1-induced CMT2 mouse model for their efficiency to restore the motor and sensory problems in these mice. In this study, we used ACY-738 and ACY-775, 2 recently reported small molecules acting as HDAC6 inhibitors in the context of an antidepressant therapy [25]. Additionally, we also tested ACY-1215 (also known as ricolinostat), which has been demonstrated to be a potent and selective HDAC6 inhibitor in human multiple myeloma cell lines [26]. ACY-1215 is active in the peripheral nervous system and was shown to work synergistically in combination with bortezomib as a treatment against multiple myeloma [26].

## Materials and Methods

### Cell Culture

Mouse neuroblastoma (N2a) cells (ATCC CCL-131) were grown in a 1:1 mixture of Dulbecco's modified eagle medium and F12 medium supplemented with glutamax (Thermo Fisher Scientific Inc., Pittsburgh, PA, USA), 100 μg/ml streptomycin, 100 U/ml penicillin (Thermo Fisher Scientific), 10% fetal calf serum (Greiner Bio-One, Kremsmünster, Austria), 1% nonessential amino acids (Thermo Fisher Scientific), and 1.6% NaHCO_3_ (Thermo Fisher Scientific) at 37°C and 7.5% CO_2_. To split the cells, cells were washed with Versene (Thermo Fisher Scientific) and dissociated with 0.05% Trypsin-EDTA (Thermo Fisher Scientific).

DRG neurons were cultured from 12-month-old Thy1.2-HSPB1 S135F mice. The DRG neurons were dissected from the spinal cord and kept in cold Hibernate A (Thermo Fisher Scientific) supplemented with B27 (Thermo Fisher Scientific), further referred to as HA/B27. To extract the DRG neurons, the tissue was dissociated by 45 min incubation of 2 mg/ml papain and thorough resuspension until all tissue was digested. The cell suspension was added to a gradient solution containing 4 layers of 37%, 25%, 20%, and 15% Optiprep (Sigma-Aldrich, St. Louis, MO, USA) in HA/B27, and centrifuged for 15 min at 800 × g (without brake), after which the cell suspension was washed in HA/B27. An additional centrifugation step was performed for 4 min at 400 × g and the cells were plated in culture medium containing Neurobasal A (Thermo Fisher Scientific) supplemented with B27 (Thermo Fisher Scientific), 200 mM L-glutamine (Thermo Fisher Scientific), 68 ng/ml nerve growth factor (Millipore, Bedford, MA, US), and antibiotics.

The N2a cells and DRG neurons were treated overnight at 37°C with dosages ranging from 10 nM to 2.5 μM of the compounds or the same volume (v/v) of dimethylsulfoxide (Sigma-Aldrich) for highest drug concentration tested.

### Western Blot Analysis

Treated cells were washed with phosphate-buffered saline (PBS) and collected using the EpiQuik Total Histone Extraction Kit (EpiGentek, Farmingdale, NY, USA) according to the manufacturer’s instructions. Protein concentrations were determined using the microBCA kit (Thermo Fisher Scientific) according to the manufacturer’s instructions. Before separating the samples on a 12% sodium dodecyl sulfate–polyacrylamide electrophoresis gel, samples containing equal amounts of protein were supplemented with reducing sample buffer (Thermo Fisher Scientific) and heated at 95°C for 5 min. After electrophoresis, the proteins were transferred to a polyvinylidene difluoride membrane (Merck Millipore, Darmstadt, Germany). The nonspecific binding was blocked by incubation of the membrane in 5% bovine serum albumin (BSA), diluted in Tris-Buffered Saline Tween (50 mM TRIS, 150 mM NaCl, 0.1% Tween-20; Applichem, Darmstadt, Germany) for 1 h at room temperature followed by incubation with primary antibodies overnight. The antibodies, diluted in Tris-Buffered Saline Tween, were directed against α-tubulin (1/5000, T6199; Sigma-Aldrich), acetylated α-tubulin (1/5000, T6793 monoclonal; Sigma-Aldrich), glyceraldehyde 3-phosphate dehydrogenase [1/1000, AM4300 (Ambion, Carlsbad, CA, USA), histone H3 acetyl k9+k14 (1/1000, 9677L, Cell Signaling, Danvers, MA, USA), and histone 4 (1/1000, ab10158; Abcam, Cambridge, UK)]. The secondary antibodies, coupled to alkaline phosphatase (anti-mouse or anti-rabbit, 1/5000; Sigma-Aldrich), were used to detect the signal of the primary antibodies. Blots were visualised by adding the enhanced chemical fluorescence substrate (GE Healthcare, Uppsala, Sweden) and imaged with the ImageQuant LAS 4000. A mild reblotting buffer (Millipore) was applied to strip the blots. ImageQuant TL version 7.0 software was used to quantify the blots.

### Immunocytochemistry

The cells were fixed by applying 4% paraformaldehyde solution for 20 min and permeabilized with 2 mg/ml BSA (Serva, Heidelberg, Germany) diluted in 0.2% Triton X-100/PBS for 10 min on ice. Nonspecific binding was blocked by 3% BSA diluted in 0.02% Triton X-100/PBS (blocking solution) for 30 min. Primary and secondary antibodies were diluted in blocking solution. The following primary antibodies, dilutions. and incubation times were used: antiacetylated α-tubulin (1/5000, T6793 monoclonal; Sigma-Aldrich), antihistone H3 acetyl k9+k14 (1/1000, 9677L; Cell Signaling), and antineuronal class III β-tubulin (802001, 1/500, overnight; BioLegend, San Diego, CA, USA). The following secondary antibodies, corresponding dilutions, and incubation times were used: donkey antimouse IgG (Alexa fluorophore-conjugated, 1/5000, 1 h; Life Technologies, Carlsbad, CA, USA) or donkey anti-rabbit IgG (Alexa fluorophore-conjugated, 1/5000, 1 h; Life Technologies). After subsequent washing with 1.5% BSA in 0.02% Triton X-100/PBS, the cover slips were mounted with 4,6-diamidino-phenylindole-containing Vectashield (Vectorlabs, Burlingame, CA, USA) to visualize the nuclei. Fluorescent micrographs were taken using a Zeiss Axio Imager M1 microscope (Carl Zeiss, Oberkochen, Germany) equipped with an AxioCam MRm3 camera (Carl Zeiss).

### Mice

All animals were housed under standard conditions according to the guidelines of the University of Leuven (KU Leuven) and the associated European guidelines (European Union Directive 2010/63/EU for animal experiments). All mice were bred to heterozygous state. All animal experiments were approved by the local ethical committee of the KU Leuven (P033/2014 and P185/2012).

Genotyping of the transgenic animals was done using KAPA Taq Ready Mix (Kapa Biosystems, Wilmington, MA, USA) and 2 sets of primers directed against the human cDNA of HSPB1 (forward primer 5’-CAGCTGGCTGACCTGTAGC-3’; reverse primer 5’-CTTGGCGGCAGTCTCATCG-3’) or directed against the mouse interleukin-2 gene, used as an internal control (forward primer 5’-CTAGGCCACAGAATTGAAAGATCT-3’; reverse primer 5’-GTAGTGGAAATTCTAGCATCATCC‐3’).

### Axonal Transport Measurements

Prior to the measurements, the cultured DRG neurons were loaded with MitoTracker Red FM (50 nM; Invitrogen, Carlsbad, CA, USA). To visualize the mitochondria, the MitoTracker was excited at 580 nm by a TILL Poly V light source (Gräfelfing, Germany). During the measurements, the DRG neurons were perfused in HEPES solution (containing 148 mM NaCl, 5 mM KCl, 0.1 mM MgCl_2_•1.6H_2_O, 10 mM glucose, 10 mM HEPES, and 2 mM CaCl_2_•2.2H_2_O) at 37°C by a heated gravity-fed perfusion system (Multichannel systems, Reutlingen, Germany), and 200 images were recorded at 1 Hz by a cooled charge-coupled device camera (Sensicam QE, PCO, Kelheim, Germany) using TILL VisION. To analyze different parameters of mitochondrial movement, kymographs, or spatiotemporal maps were obtained using Igor Pro (Wavemetrics, Portland, USA) using custom-written routines based on a previous described analysis algorithm [27].

### Electrophysiology

For the nerve-conduction measurements, mice were anesthetized using 1% isoflurane/O_2_ gas inhalation and placed on a heating pad to maintain body temperature. Using subdermal needle electrodes (Technomed Europe, Maastricht, the Netherlands) and a Medelec Syngery EMG monitor (Vickers Medical, Sidcup, UK) compatible with Synergy software (version 20.1.0.100), the CMAPs were assessed by supramaximal stimulation (1 pulse/s, 0.1 ms stimulus duration) at the level of the sciatic notch and recording at the gastrocnemius muscle [12]. SNAPs were recorded by supramaximal stimulation (6 pulse/s, 0.1 ms stimulus duration) at the distal tip of the tail and were measured 4 cm proximal in the tail [28]. For SNAP recordings, multiple traces were averaged.

### Histological Examination of Neuromuscular Junctions

Prior to dissection, the mice were euthanized by CO_2_ inhalation and subsequent cervical dislocation. The gastrocnemius muscle was dissected and snap-frozen in liquid nitrogen-cooled isopentane. Cryosections (20 μm) were fixed with 4% paraformaldehyde for 10 min and rinsed with PBS. Aspecific binding was blocked using 5% normal donkey serum (Sigma-Aldrich) diluted in PBS-0.1% Triton X-100. Nerve axons were visualized using antineurofilament light-chain antibody (Alexa488 conjugated, 8024S, 1/500, overnight incubation; Cell Signalling) while the neuromuscular endplate was labeled with α-bungarotoxin (Alexa555-conjugated, 1/5000, 1 h incubation; Life Technologies). To quantify the number of innervated neuromuscular junctions (NMJs), a least 100 junctions from each muscle was checked for colocalization of the neurofilament light chain and α-bungarotoxin signal. Fluorescent micrographs were taken using a Zeiss Axio Imager M1 microscope (Carl Zeiss) equipped with an AxioCam MRm3 camera (Carl Zeiss).

## Results

In this study, we used ACY-738 and ACY-775, pyrimidine hydroxyl amide small-molecule inhibitors that were previously profiled for their pharmacokinetic properties and that showed nanomolar potency and selectivity for HDAC6 over other HDACs [25]. In Table 1 , the most important molecular and pharmacokinetic properties are summarized [25]. The most potent compound is ACY-738 with an IC_50_ value towards HDAC6 of 1.7 nM, while ACY-775 is the most selective inhibitor (selectivity index HDAC1/HDAC6 of 283). The third molecule, ACY-1215, was chosen not only for its potency and selectivity towards HDAC6 [26], but also because this compound is currently tested in a phase II clinical trial for multiple myeloma (clinicaltrails.gov identifier: NCT01323751) [26]. Hence, positive results in this CMT2-based screening could facilitate the translation of ACY-1215 to the clinic as a therapy for CMT2.

**Table 1:**
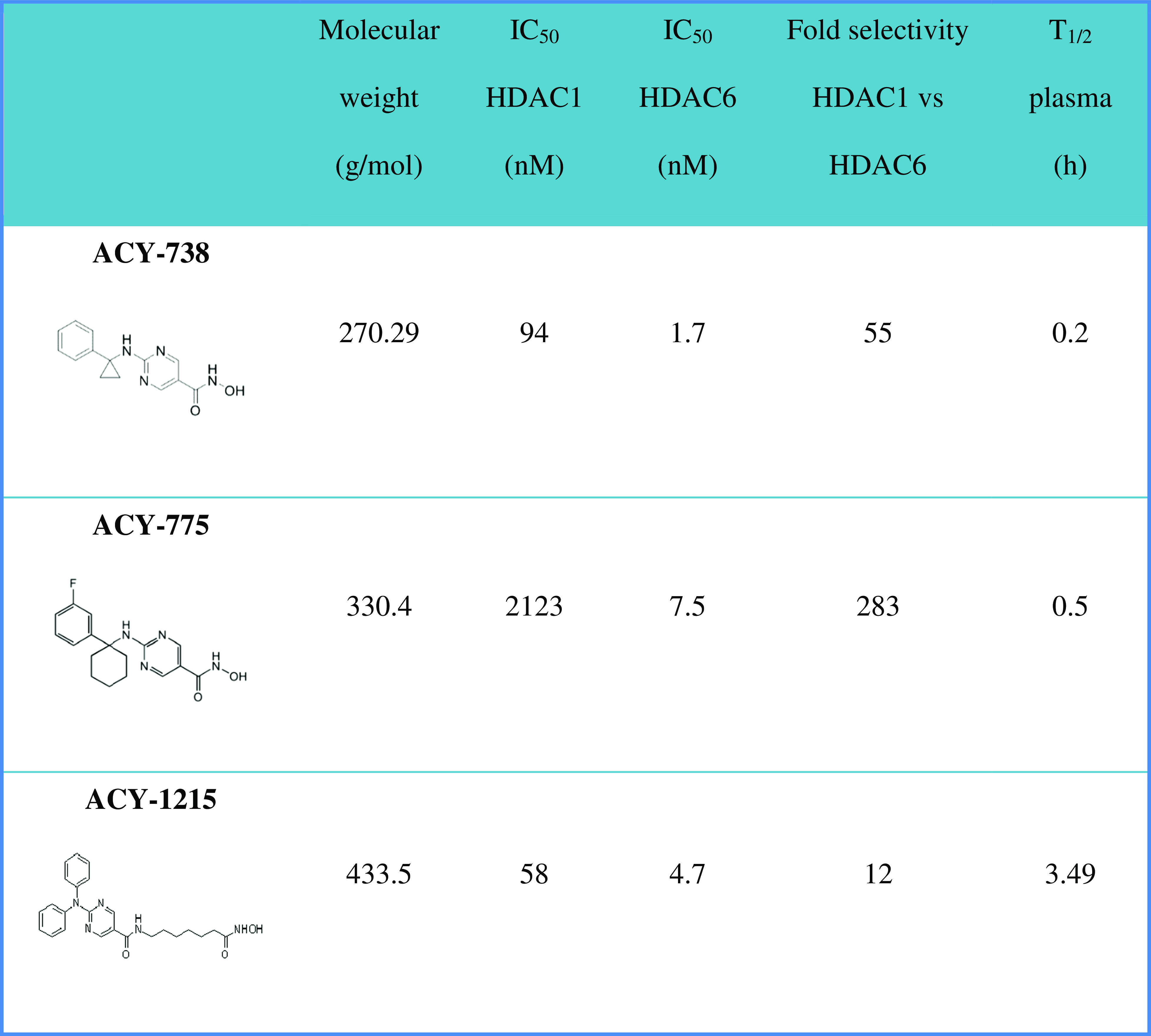
Molecular and pharmacokinetic properties of ACY-738, ACY-775, and ACY-1215 as obtained from Jochems et al. [25]

HDAC = histone deacetylase

### ACY-738 and ACY-775 are Potent and Selective HDAC6 Inhibitors in N2a Cells

**Fig. 1:**
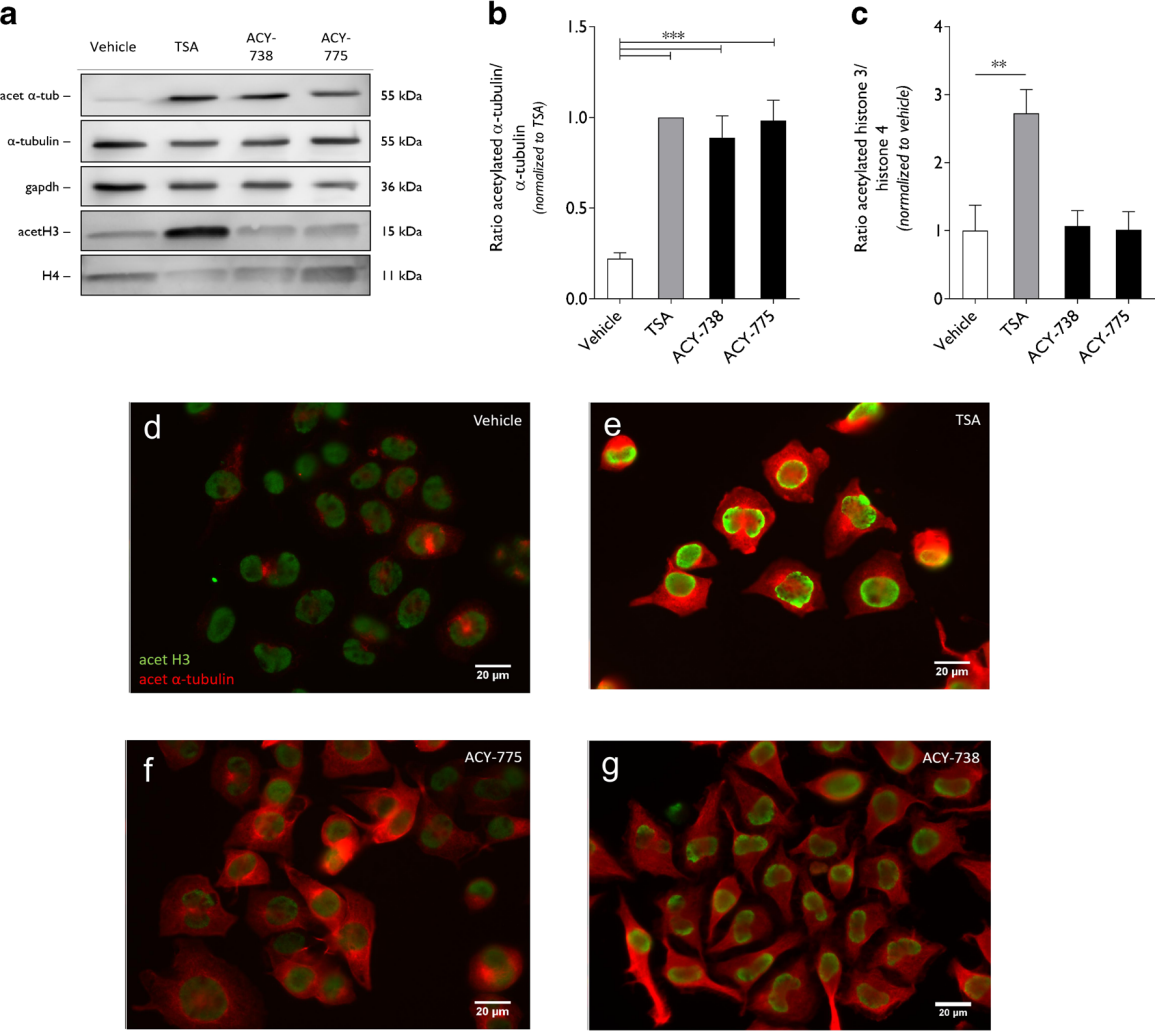
ACY-738 and ACY-775 are potent and selective histone deacetylase 6 inhibitors. (**a**) N2a cells were incubated with 1 μM ACY-738, ACY-775, or trichostatin A (TSA) as a positive control. By Western blot the acetylation of α-tubulin (acet α-tub) was assessed, as well as the acetylation of histone 3 (acetH3). Total α-tubulin levels, glyceraldehyde 3-phosphate dehydrogenase (gapdh) and histone 4 (H4) were used as a reference for equal loading. (**b**) The ratio of acetylated α-tubulin to total tubulin levels was calculated and normalized to TSA-treated cells. One-way analysis of variance (ANOVA); ***p < 0.0001; *n* = 4. (**c**) The ratio of acetylated histone 3 to histone 4 levels was assessed and subsequently normalized to vehicle-treated cells. One-way ANOVA; **p < 0.01; *n* = 3–4. (**d**–**g**) Immunocytochemistry for acetylated α-tubulin (red) and acetylated histone 3 (green) on N2a cells treated with 1 μM TSA, ACY-738, and ACY-775

In the cellular context of N2a cells, the effect of ACY-738 and ACY-775 on the acetylation status of α-tubulin and histone 3 was assessed by Western blot (Fig. 1a). As a pan-HDAC inhibitor, TSA enhances the acetylation of both α-tubulin and histone 3, and therefore was used as a positive control in this screening (Fig. 1a–c). The HDAC6 inhibitors, ACY-738 and ACY-775, hyperacetylated α-tubulin at a concentration of 1 μM. The histone acetylation was not affected (Fig. 1a–c). Immunocytochemistry was used to visualize the acetylation of α-tubulin, which is present in the cytoplasm, and of histone 3, visible in the nucleus. In vehicle-treated cells, α-tubulin was mainly present in the deacetylated form, while histone 3 was clearly acetylated (Fig. 1a, d). Upon treatment with ACY-738 and ACY-775, a clear enhancement of the acetylation of α-tubulin was visible, while histone acetylation remained unaltered (Fig. 1f–g).

**Fig. 2:**
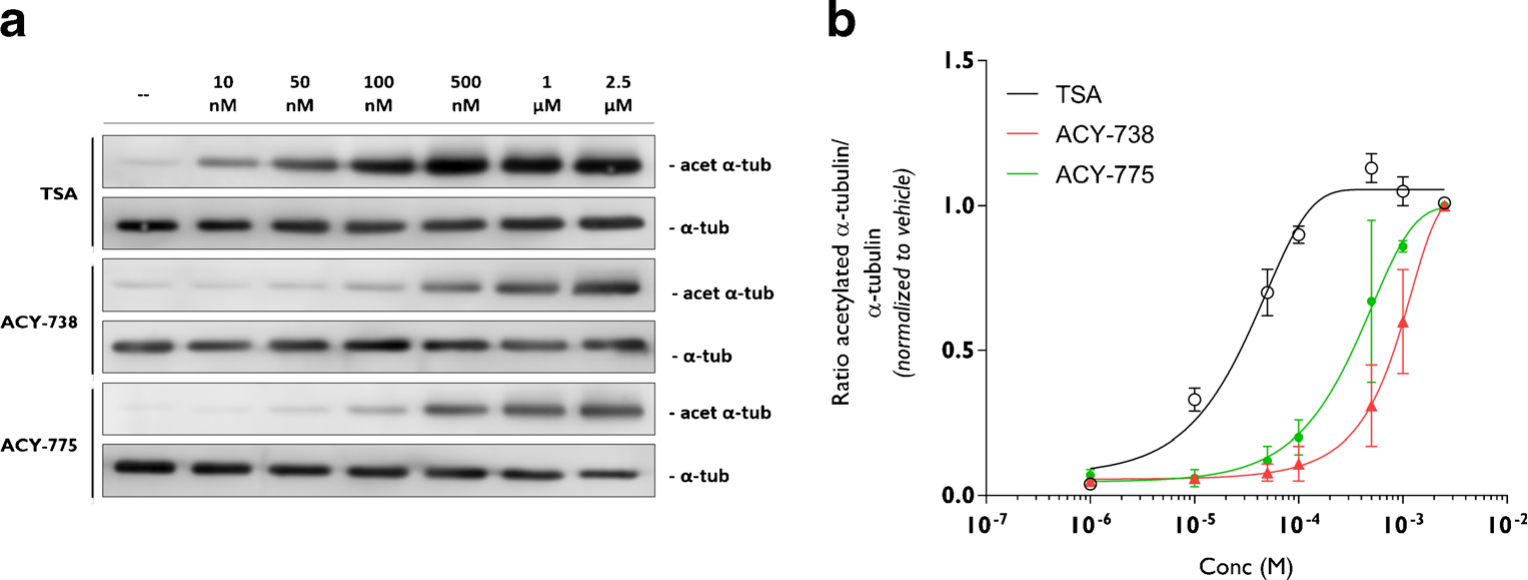
Pharmacokinetic properties of ACY-738 and ACY-775 in N2a cells. (**a**) Dose–response of trichostatin A (TSA), ACY-738, or ACY-775 on acetylation of α-tubulin was established in N2a cells and determined by Western blot. (**b**) Quantification of the ratio of acetylated α-tubulin to total tubulin levels on Western blot. Values were normalized to the effect of 2.5 μM (*n* = 3)

A more profound insight into the pharmacokinetic properties of ACY-738 and ACY-775 on the acetylation of α-tubulin was obtained by establishing a dose–response curve in N2a cells using concentrations ranging from 10 nM up to 2.5 μM. Both ACY-738 and ACY-775 possessed a similar stimulating activity towards α-tubulin acetylation (Table 1, Fig. 2a). At a concentration of 100 nM, the acetylation of α-tubulin was enhanced, reaching saturating levels at 1 μM. TSA reached hyperacetylation of α-tubulin at 100 nM (Fig. 2a, b). This is in contrast to ACY-738 and ACY-775, which required a higher concentration to reach their maximal effect (Fig. 2a, b).

### Mitochondrial Transport Defects are Rescued by HDAC6 Inhibitors ACY-738 and ACY-775

**Fig. 3:**
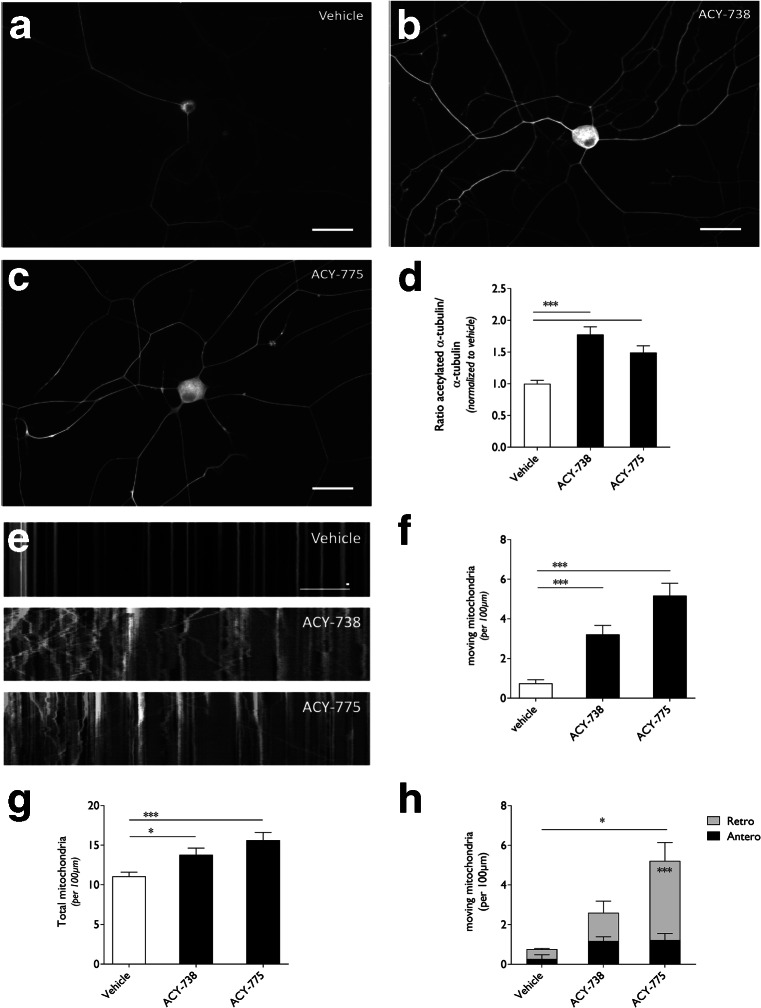
Histone deacetylase 6 (HDAC6) inhibition using ACY-738 and ACY-775 rescued the mitochondrial defects in dorsal root ganglion (DRG) neurons cultured from symptomatic HSPB1^S135F^ mice. (**a**–**c**) Nontransgenic DRG neurons were incubated with 2.5 μM ACY-738 or ACY-775. Immunocytochemical labeling of acetylated α-tubulin shows increased immunofluorescence in neurons upon HDAC6 inhibition. Scale bar = 40 μm. (**d**) The intensity of acetylated α-tubulin was quantified in the neurites from DRG neuron cultures and corrected for the length of the signal. All values within each experiment were normalized to vehicle-treated cells; *n* = 5 with 43–61 cells per condition. (**e**) Kymographs representing the axonal movement of mitochondria in HSPB1^S135F^ DRG neurons treated with ACY-738 or ACY-775 (2.5 μM). Vertical lines representing stationary mitochondria and lines deflecting to the right or left indicate anterograde or retrograde movement of mitochondria, respectively. (**f**) The number of moving mitochondria per 100 μm in the neurites was quantified based on the kymographs from HSPB1^S135F^ DRG neurons treated with ACY-738 or ACY-775; *n* = 25–35 from 3 different transgenic mice. (**g**) The total number of mitochondria per 100 μm in the neurites was quantified based on the kymographs from HSPB1^S135F^ DRG neurons treated with ACY-738 or ACY-775; *n* = 25–35 from 3 different transgenic mice. (**h**) Additional analysis was performed to assess the number of anterogradely or retrogradely moving mitochondria per 100 μm in the neurites based on the kymographs from HSPB1^S135F^ DRG neurons treated with ACY-738 or ACY-775; *n* = 3 with 7–10 cells per experiment. One-way analysis of variance (ANOVA); *p < 0.05, ***p < 0.0001. Two-way ANOVA; *p < 0.05, ***p < 0.0001

ACY-738 and ACY-775 were subsequently tested for their ability to restore the mitochondrial axonal transport defects seen in DRG neurons cultured from symptomatic HSPB1^S135F^ mice. To assess whether ACY-738 or ACY-775 could affect acetylation of α-tubulin in primary neuronal cells, DRG neurons were cultured from adult nontransgenic mice (NTG) and treated overnight with the HDAC6 inhibitors (Fig. 3a–c). Acetylation of α-tubulin was visualized by immunofluorescence (as shown in Fig. 3a–c) and the intensity in the neurites of the neurons was quantified and normalized to the length of the fluorescent signal (Fig. 3d). In vehicle-treated DRG neurons, acetylated α-tubulin was already present (Fig. 3a). Upon treatment with ACY-738 or ACY-775 the signal intensity of acetylated α-tubulin increased significantly (Fig. 3d).

Next, DRG neurons were cultured from symptomatic HSPB1^S135F^ mice and treated with the highest dose (2.5 μM) tested in NTG DRG neurons to investigate the ability of ACY-738 and ACY-775 to restore the mitochondrial axonal transport defects. Kymographs represent the general movement of mitochondria in the neurites of HSPB1^S135F^ DRG neurons treated with vehicle, ACY-738 or ACY-775 (Fig. 3e). Significant increased motility of mitochondria and also the total number of mitochondria within the neurites was observed compared with vehicle-treated DRG neurons (Fig. 3f, g). Interestingly, a significantly higher number of retrogradely transported mitochondria was observed in DRG neurons treated with ACY-775 compared with vehicle-treated cells (Fig. 3h).

### ACY-738, ACY-775, and ACY-1215 Reverse the Motor and Sensory Axonal Deficits in HSPB1^S135F^ CMT2 Mouse Model

The last phase of the screening aimed to establish the effect of HDAC6 inhibition by ACY-738 or ACY-775 on the motor and sensory deficits of the HSPB1^S135F^-induced CMT2 mouse model. We included an additional HDAC6 inhibitor, ACY-1215, in this assay as it was characterized previously and as it is currently in clinical trials [29]. These experiments are also an indirect measure of the pharmacodynamic properties of the different compounds.

**Fig. 4:**
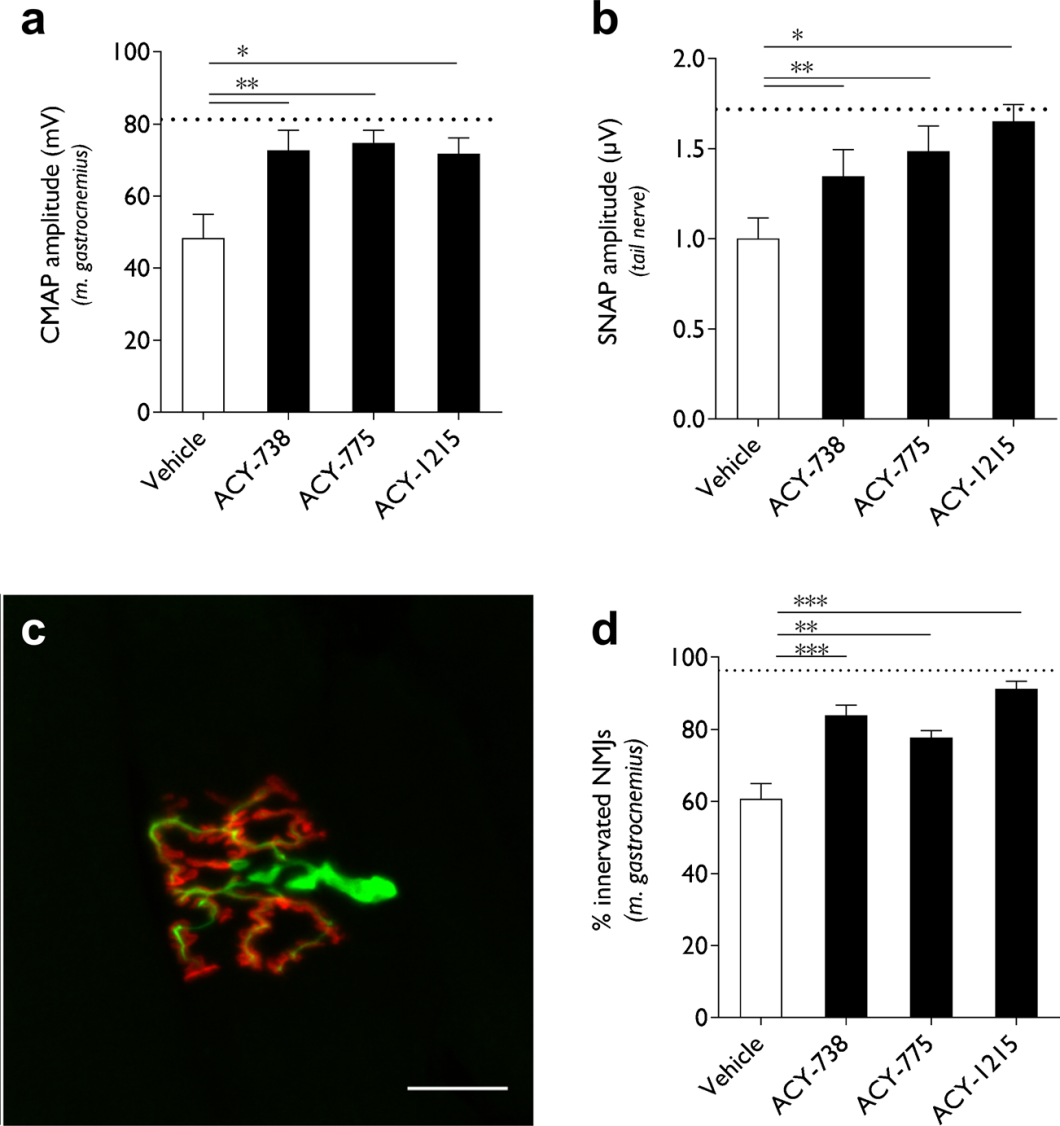
Histone deacetylase 6 (HDAC6) inhibition using ACY-738, ACY-775, or ACY-1215 reversed the axonal deficits in motor and sensory nerves and induced reinnervation of neuromuscular junction. (**a**) Symptomatic (12-month-old) HSPB1S135F mice received daily intraperitoneal injections of ACY-738, ACY-775 (3 mg/kg), ACY-1215 (30 mg/kg), or vehicle for 21 days. The compound muscle action potential (CMAP) amplitudes were recorded from the sciatic nerve after the treatment period. Dashed line indicates values obtained from nontransgenic mice (NTG) mice; *n* = 5–8 mice. (**b**) The SNAP amplitudes recorded over the sensory tail nerve after the treatment. Dashed line indicates values obtained from NTG mice; *n* = 5–8 mice. Dunnett's multiple comparison test, *p < 0.05, **p < 0.01, ***p < 0.001. (**c**) After treatment, the neuromuscular junctions (NMJs) were visualized from the gastrocnemius muscle by immunofluorescence. The motor endplates were labeled by α-bungarotoxin and the axon terminals were visualized by neurofilament light chain-directed antibody. Dashed line indicates values obtained from NTG mice. (**d**) The number of innervated NMJs was quantified by determining the percentage of overlap between α-bungarotoxin and neurofilament light chain immunofluorescence. Dashed line indicates values obtained from NTG mice; *n* = 5–8 mice. Dunnett's multiple comparison test, ** *p* <0.01, ***p < 0.001

Symptomatic HSPB1^S135F^ mice received daily intraperitoneal injections of ACY-738, ACY-775, or ACY-1215 for 21 days, after which nerve-conduction studies were performed in motor and sensory nerve nerves. Vehicle-treated mice displayed reduced CMAP and SNAP amplitudes measured in the sciatic nerve and tail nerve, respectively (Fig. 4a, b). The HDAC6 inhibitors, ACY-738, ACY-775, were administered at 3 mg/kg which is an 8× lower dosage than in the original study using tubastatin A [13]. ACY-1215 was tested at a higher dose (30 mg/kg). All inhibitors increased the action potential amplitudes in motor and sensory nerves (Fig. 4a, b). After treatment, the mice were sacrificed and the gastrocnemius muscle was dissected to visualize reinnervation of the NMJs. The motor endplate was labeled using α-bungarotoxin, while the axonal projections were identified using an antibody recognizing neurofilament light chain. Quantifying the number of double-labeled NMJs demonstrated a significant increase in the innervation of the gastrocnemius muscle upon treatment with all three HDAC6 inhibitors (Fig. 4c, d).

## Discussion

The main goal of this study was to accelerate the translation of the beneficial effects of HDAC6 inhibition seen in a CMT2 mouse model [13] into a pharmacological therapy for patients with CMT for which currently no treatment exists. Three pyrimidine hydroxyl amide small molecules (ACY-738, ACY-775, ACY-1215) were tested in a screening method based on the behavioral and pathological abnormalities of the HSPB1^S135F^-induced CMT2 mouse model [13]. The chemical structures of the tested compounds all contained a similar functional group, which classifies them as hydroxamic acids, and only differed in the capping group. So far, 3 small molecules harboring a hydroxamic acid (vorinostat, panobinostat, and belinostat) have been approved as anticancer agents and these compounds are all broad-spectrum HDAC inhibitors [30]. However, these compounds exhibit poor pharmacokinetics, including rapid metabolic degradation, and poor selectivity, inducing off-target effects and toxicity [30]. As a consequence, selective HDAC inhibitors with improved pharmacokinetic and pharmacodynamic properties are urgently needed.

In the first phase of our study, ACY-738 and ACY-775 demonstrated selective and potent inhibition towards HDAC6. Both HDAC6 inhibitors were able to rescue the axonal transport defects of mitochondria seen in cultured DRG neurons from a mutant HSPB1–CMT2 mouse model. ACY-775 showed a higher potency to induce α-tubulin acetylation than ACY-738, as indicated by the larger effect on the mitochondrial transport defects, despite the higher IC_50_ value of ACY-775. Most importantly, ACY-738 and ACY-775 could also reverse the deficits observed in the motor and sensory nerves of the CMT2 mouse model. Interestingly, symptomatic HSPB1^S135F^ mice only show axonal loss in the distal part of the sciatic nerve [13]. Therefore, we examined the innervation of NMJs in the gastrocnemius muscle after treatment with HDAC6 inhibitors. The enhanced CMAP amplitudes measured in the gastrocnemius muscle after treatment is in line with histological evidence of improved innervation of the NMJs. On the sensory side, histopathology of the sensory nerve endings using immunofluorescent labeling of PGP9.5, as well as β3-tubulin, was performed in transverse sections of the skin of the tail, looking at the intraepidermal nerve fiber densities. So far, we could not observe any morphological structures and thus further investigation is needed to determine the effect of HDAC6 inhibition on the sensory nerve regeneration in relation to the improved SNAP amplitudes.

In addition, we could confirm the positive effects of ACY-738 and ACY-775 using ACY-1215. This selective HDAC6 inhibitor has already been extensively characterized with regard to its enzymatic potency and selectivity towards HDACs. We observed a significant effect of ACY-1215 on both the motor and sensory defects in CMT2 mice. Currently, ACY-1215 is tested in a phase II clinical trial for multiple myeloma, which makes it a convenient lead molecule to be tested as a pharmacological treatment in patients with CMT. Hence, these positive results in this CMT2-based screening can help to advance the translation of ACY-1215 into a new CMT2 therapy.

ACY-738, ACY-775, and ACY-1215 are all hydroxamic-based structures. Despite the widespread use of hydroxamic acids as HDAC inhibitors, concerns have been raised about the potential mutagenicity of hydroxamic acids. These compounds could have the ability to induce point mutations, insertions, or deletions in DNA, chromosomal breakages, and/or the rearrangement of chromosomes [30]. Molecules having this tendency are thus potentially carcinogenic. Nevertheless, ACY-1215 has been shown to be safe and well tolerated in a phase I study and to be Ames-negative. As a consequence, this HDAC6 inhibitor is an attractive molecule to treat patients with CMT.

Pharmacologic inhibition of HDAC6 is a promising new therapeutic target for patients with CMT and our findings add to the growing list of ongoing preclinical and clinical research. However, it remains to be demonstrated whether the therapeutic effect of HDAC6 inhibition is only relevant for patients with a mutation in HSPB1, or whether it can be extrapolated to other forms of CMT2, or even to CMT1. At present, most pharmacologic strategies focused on this demyelinating form of CMT. Unfortunately, the beneficial effects of ascorbic acid observed in mouse models of CMT1 could not be reproduced in patients with CMT1A [31–34]. The effect of neurotrophin-3 administration on myelination and on the peripheral neuropathies needs further investigation before in can be translated to the clinic [35]. The most promising therapeutic strategy at this moment is the combinatorial therapy consisting of baclofen, naltrexone, and sorbitol (PXT3003), which improved myelination and nerve conduction in a CMT1A preclinical study and was shown to be safe and well tolerated in a phase II clinical trial. Currently, a phase III study is performed to determine the effectiveness of PXT3003 in patients with CMT1A [36]. Thus, further research is needed to demonstrate the therapeutic potential of HDAC6 inhibition in CMT2 and to add HDAC6 inhibition to the list of possible treatments for patients with CMT2.

Interestingly, inhibition of the deacetylating function of HDAC6 or increasing acetylation of α-tubulin have been shown to ameliorate axonal transport deficits in models for Parkinson’s disease and Alzheimer’s disease [11, 37]. Mitochondrial axonal transport defects are often associated with neurodegeneration in general and with peripheral neuropathies in particular [8]. Thus, investigating the effect of HDAC6 inhibition in other peripheral neuropathies could be another interesting route for further investigations.

HDAC6 inhibition significantly improved the mitochondrial axonal transport dynamics, specifically in the retrograde direction. Kinesin requires the acetylated form of α-tubulin to transport cargoes anterogradely along the microtubules [7]. However, the acetylated form of α-tubulin alone is not sufficient to affect kinesin-1 velocity and run length *in vitro* [38, 39]. Therefore, increasing α-tubulin acetylation through inhibition of the deacetylating function of HDAC6 does not imply a direct effect on anterograde transport. Alternatively, HDAC6 is known to be implicated in the recruitment of polyubiquitinated proteins to the aggresome via the dynein motor complex [40, 41]. Mutations in HSPB1 lead to the formation of cytoplasmic aggregates [17]. In our mutant HSPB1-induced CMT2 mouse model, we suggest a dual hypothesis where HDAC6 transports polyubiquitinated proteins, such as mutated HSPB1, to the aggresome but, in turn, deacetylates the microtubules causing a block of axonal transport. In our DRG neuron cultures, inhibition of HDAC6 specifically stimulated the retrograde transport of mitochondria thereby supporting our dual hypothesis. Additionally, Calderilla-Barbosa et al. [42] proposed a model in which HDAC6 and SQSTM-1, both recognizing polyubiquitinated proteins, compete for the binding to dynein and where the interaction of SQSTM-1 with dynein is necessary for proper dynein function. This could indicate that the beneficial effect of HDAC6 inhibition on retrograde transport is mediated through the dynein motor.

Acetylation of α-tubulin is thus a key component in the process of mitochondrial axonal transport and important in neurons where axonal transport is required to meet the high-energy demands at the synapses [43, 44]. Alterations in acetylation of α-tubulin have been associated with cancer, heart disease, inflammation, and immunity, and also with many neurologic disorders [44]. Additionally, HDAC6 inhibition could also improve cognitive deficits in a mouse model for Alzheimer’s disease, has also been shown to exhibit antidepressant effects, and its therapeutic effect has also been demonstrated in a model of ischemic brain infarction [25, 45, 46]. As a consequence, identifying HDAC6 inhibitors with improved pharmacokinetic and pharmacodynamic properties will not only be important for the development of a pharmacological therapy for CMT, but could also be important for a variety of other disorders.

Further investigation and optimization of our screening method is also required, as the current method resulted in a limited number of compounds that could be tested simultaneously. Several alternative techniques exist to speed up and scale up the compound screening from N2a cells to animal testing. Detection of acetylation of α-tubulin and histones by Western blot can be substituted by AlphaLisa [47], which, compared with Western blot, requires no washing steps, and harbors a larger detection window and a shorter assaying time. Technology involving induced pluripotent stem cells (iPSCs) and the differentiation toward specific cell types is rapidly advancing [48]. Moreover, the CRISPR-Cas9 technique offers a wide variety of possibilities in working with human cells [49]. Starting from CMT2 patient-derived fibroblasts, iPSC-derived DRG neurons could be used to identify the effect of new HDAC6 inhibitors on α-tubulin acetylation and mitochondrial deficits. Moreover, iPSCs can also be differentiated into motor neurons. As we can only culture embryonic motor neurons while the Thy-1.2 promoter is only active postnatally, we are not able to test the effect of HDAC6 inhibitors in motor neurons from mutant HSPB1 mice. Hence, using the iPSC-based technology, a larger number of compounds could be tested for their ability to restore the mitochondrial axonal transport defects in both motor and sensory neurons.

In conclusion, we established a screening method consisting of 3 different phases identifying selective and potent HDAC6 inhibitors that can functionally restore motor and sensory deficits in a CMT2F mouse model. One of these inhibitors has the potential to be translated into the clinic as it is already being tested in a clinical trial and as it is Ames-negative. Future research will be directed towards novel classes of HDAC6 inhibitors that carry alternative functional motifs [50]. Moreover, studies exploring the therapeutic potential of HDAC6 inhibition in other genetic forms of CMT are crucial to get an idea about the therapeutic potential of HDAC6 inhibitors in CMT.

## Electronic supplementary material

Below is the link to the electronic supplementary material.

ESM 1 (PDF 461 kb)
